# Supplementary material for: Effects of Climate Conditions before Harvest Date on Edamame Metabolome
Source: Plants (Basel). 2023 Dec 27;13(1):87. doi: 10.3390/plants13010087 (PMC10780805; doi:10.3390/plants13010087)
Supplement: Supplementary file 1 [file plants-13-00087-s001.zip › Caption for Supplementary files.pdf]

Figure S1. Climate conditions during the 3, 5, 7, and 9 days before harvesting. Each color represents data of each year. Dotted line shows the average values of each climate condition for 6 years. SD; sunshine duration, AT; average temperature, RF; rain fall. SD_x means the SD amount accumulated x days before harvest.

Figure S2. The amounts of citric acid in edamame at each harvest day for 6 years.

Figure S3. Annual fluctuation of the accumulated sunshine duration (SD) during the 1, 3 ,5, 7, and 9 days before harvesting. SD_x means the SD amount accumulated x days before harvest.

Figure S4. Annual fluctuation of the accumulated average temperature (AT) during the 1, 3 ,5, 7, and 9 days before harvesting. AT_x means the AT amount accumulated x days before harvest.

Figure S5. Annual fluctuation of the accumulated rain fall (RF) during the 1, 3 ,5, 7, and 9 days before harvesting. RF_x means the RF amount accumulated x days before harvest.

Figure S6. Annual fluctuation of the accumulated average wind speed (AWS, left) and temperature differences (TD, right) during the day before harvesting.

Figure S7. Principal component analysis using climate conditions and edamame metabolome for 6 years. The scores plot showed the cluster distribution of the harvest years along with PC1 and PC2 axis. The color of the symbol indicates each harvest year, as shown on the upper right.

Table S1. Fifty-eight types of ionic compounds quantified in edamame produced between 2013 and 2018.
