# Supplementary material for: Effects of Climate Conditions before Harvest Date on Edamame Metabolome
Source: Plants (Basel). 2023 Dec 27;13(1):87. doi: 10.3390/plants13010087 (PMC10780805; doi:10.3390/plants13010087)
Supplement: Supplementary file 1 [file plants-13-00087-s001.zip › supplemental Figures S1-S7.pptx]

## Slide 1
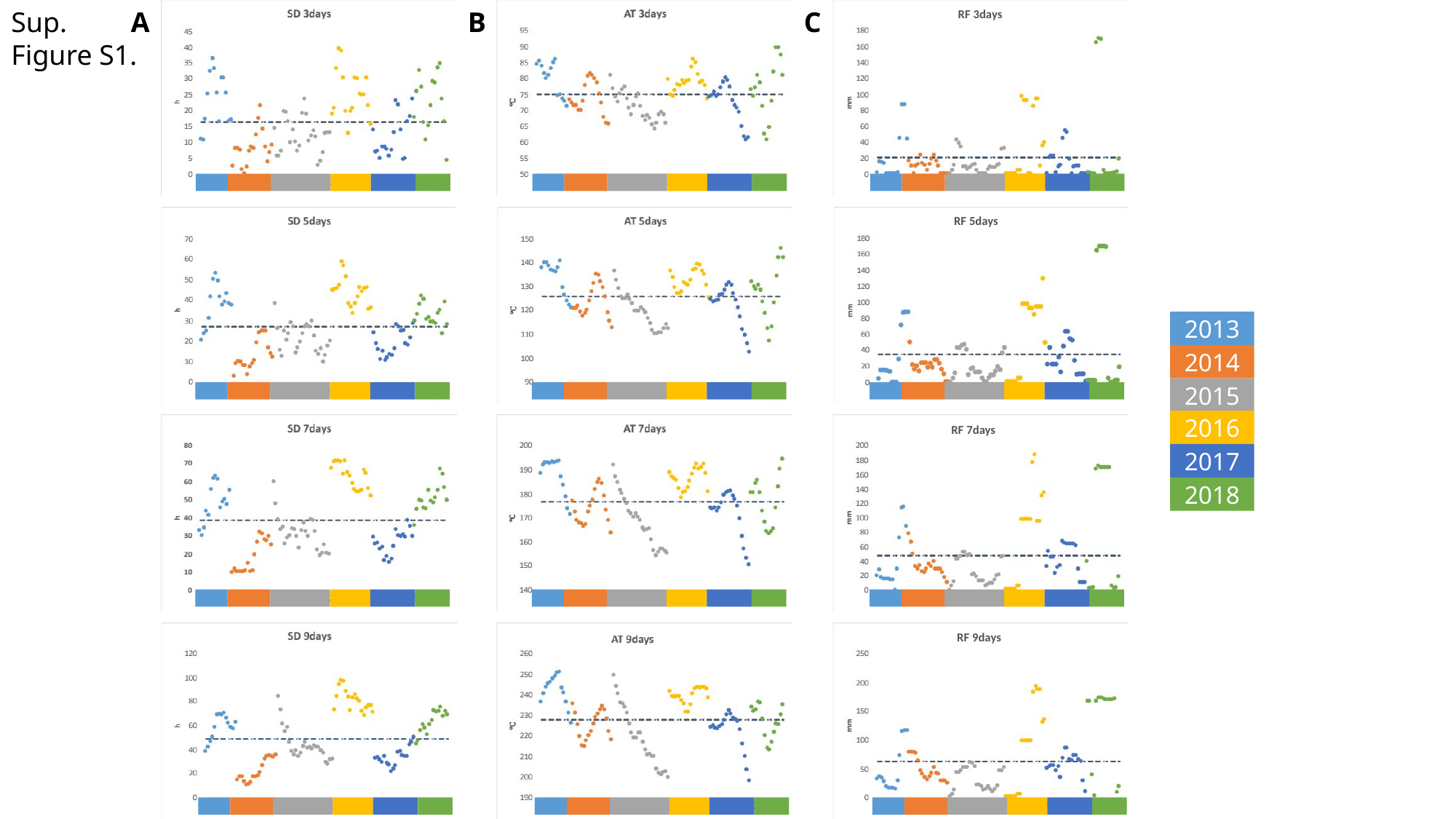

A
B
C
Sup.
Figure S1.
RF 3days
RF 5days
2013
2014
2015
2016
RF 7days
2017
2018
RF 9days

## Slide 2
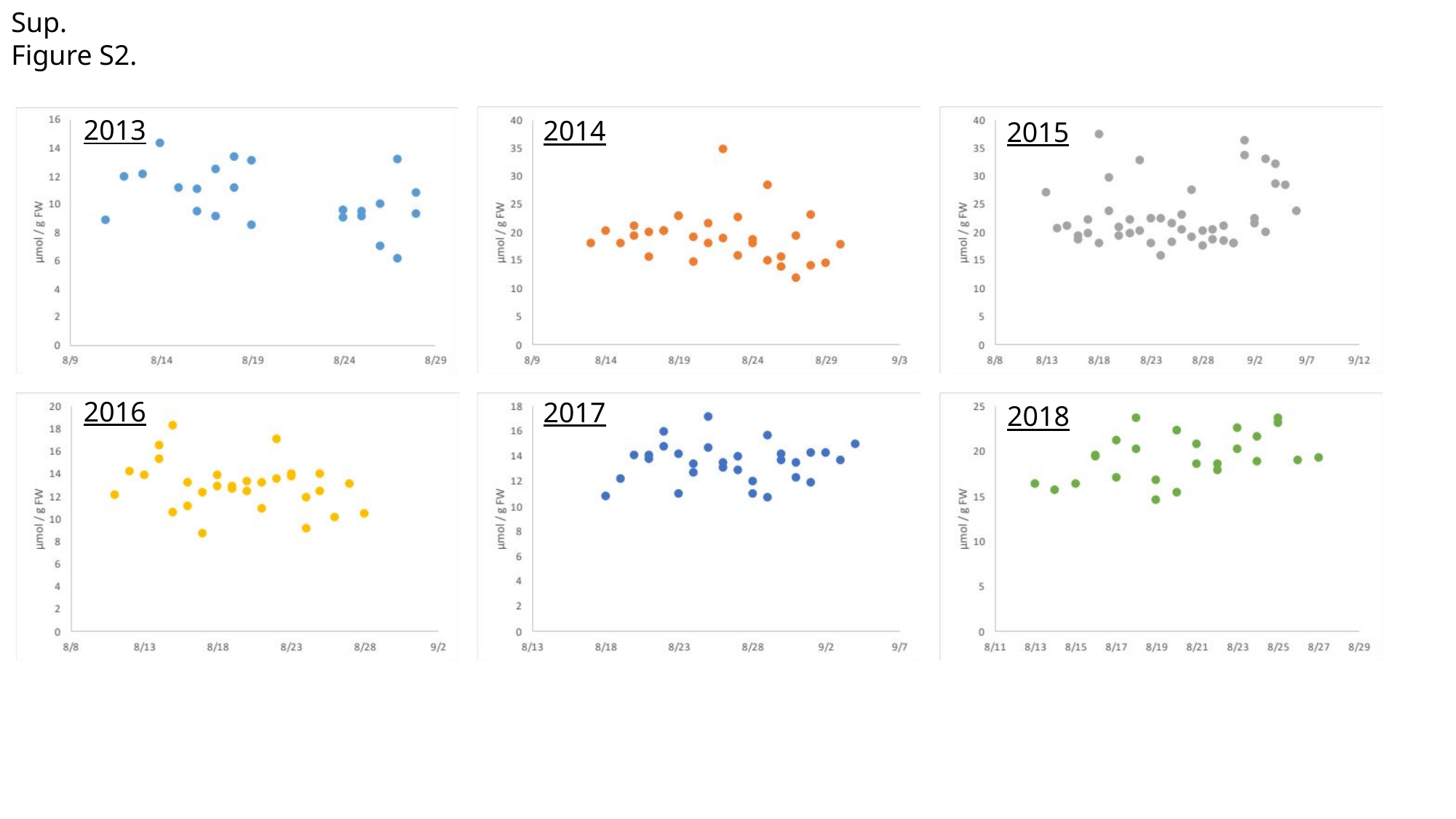

Sup.
Figure S2.
2013
2014
2015
2016
2017
2018

## Slide 3
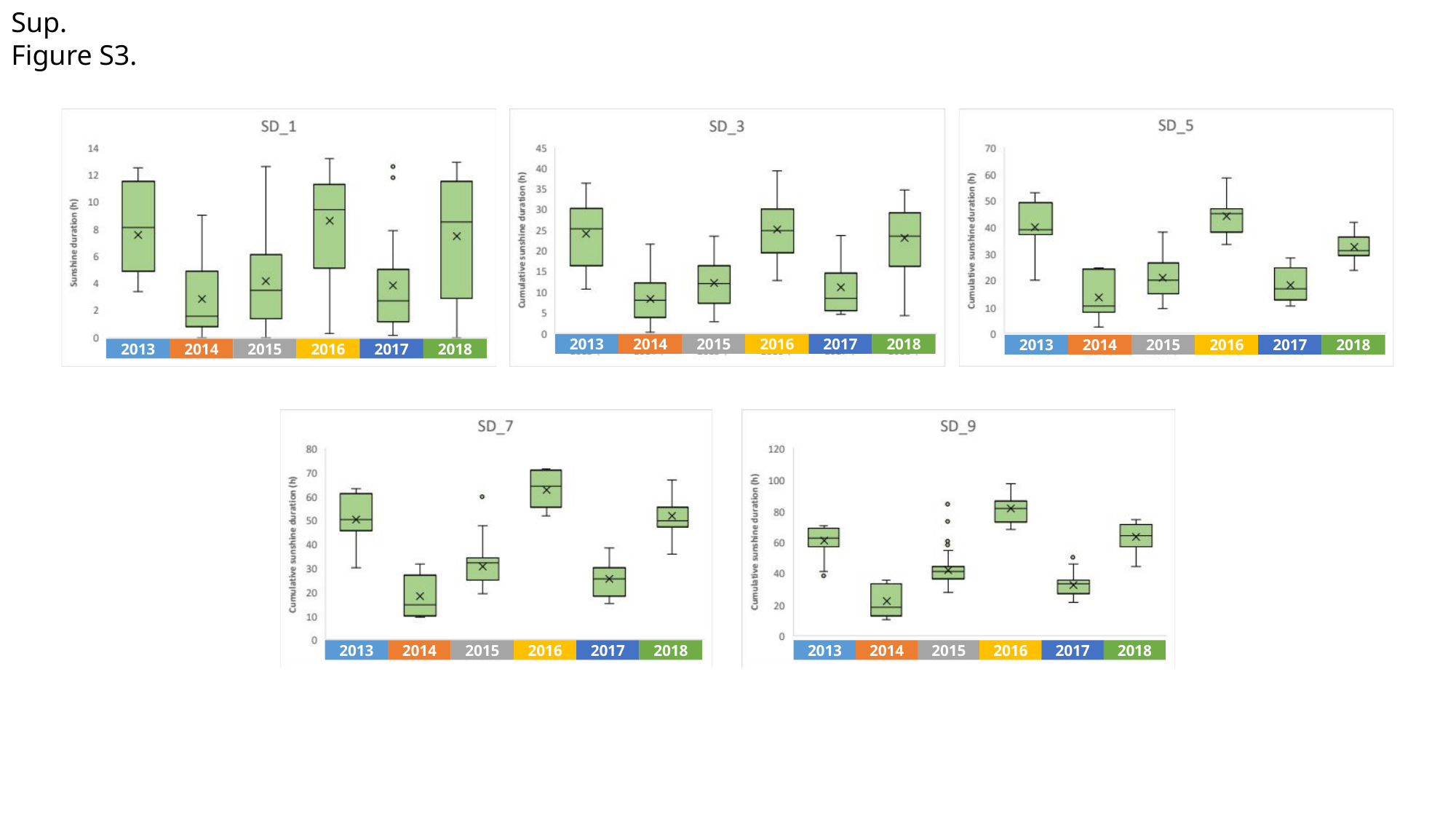

Sup.
Figure S3.
2013
2014
2015
2016
2017
2018
2013
2014
2015
2016
2017
2018
2013
2014
2015
2016
2017
2018
2013
2014
2015
2016
2017
2018
2013
2014
2015
2016
2017
2018

## Slide 4
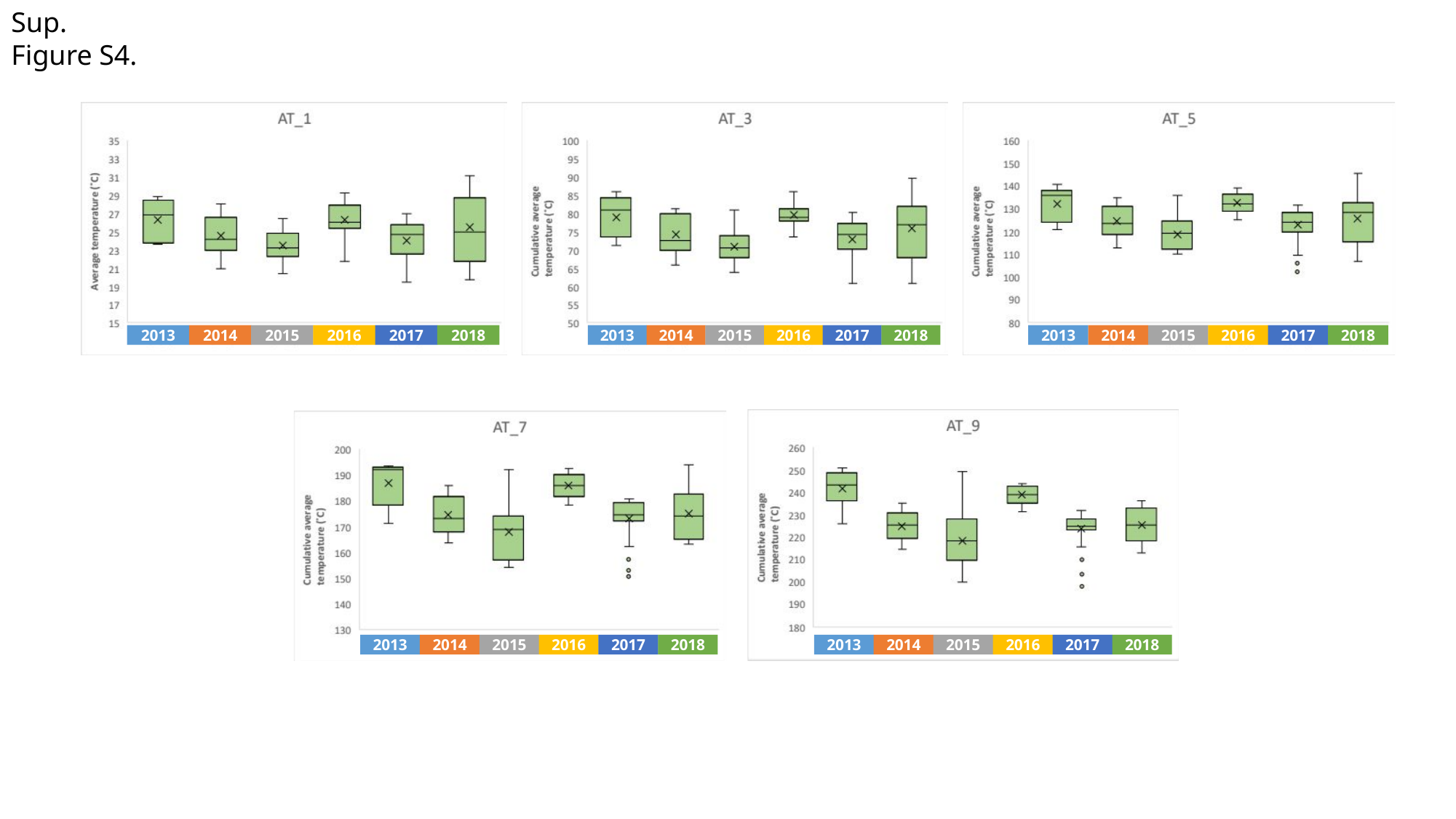

Sup.
Figure S4.
2013
2014
2015
2016
2017
2018
2013
2014
2015
2016
2017
2018
2013
2014
2015
2016
2017
2018
2013
2014
2015
2016
2017
2018
2013
2014
2015
2016
2017
2018

## Slide 5
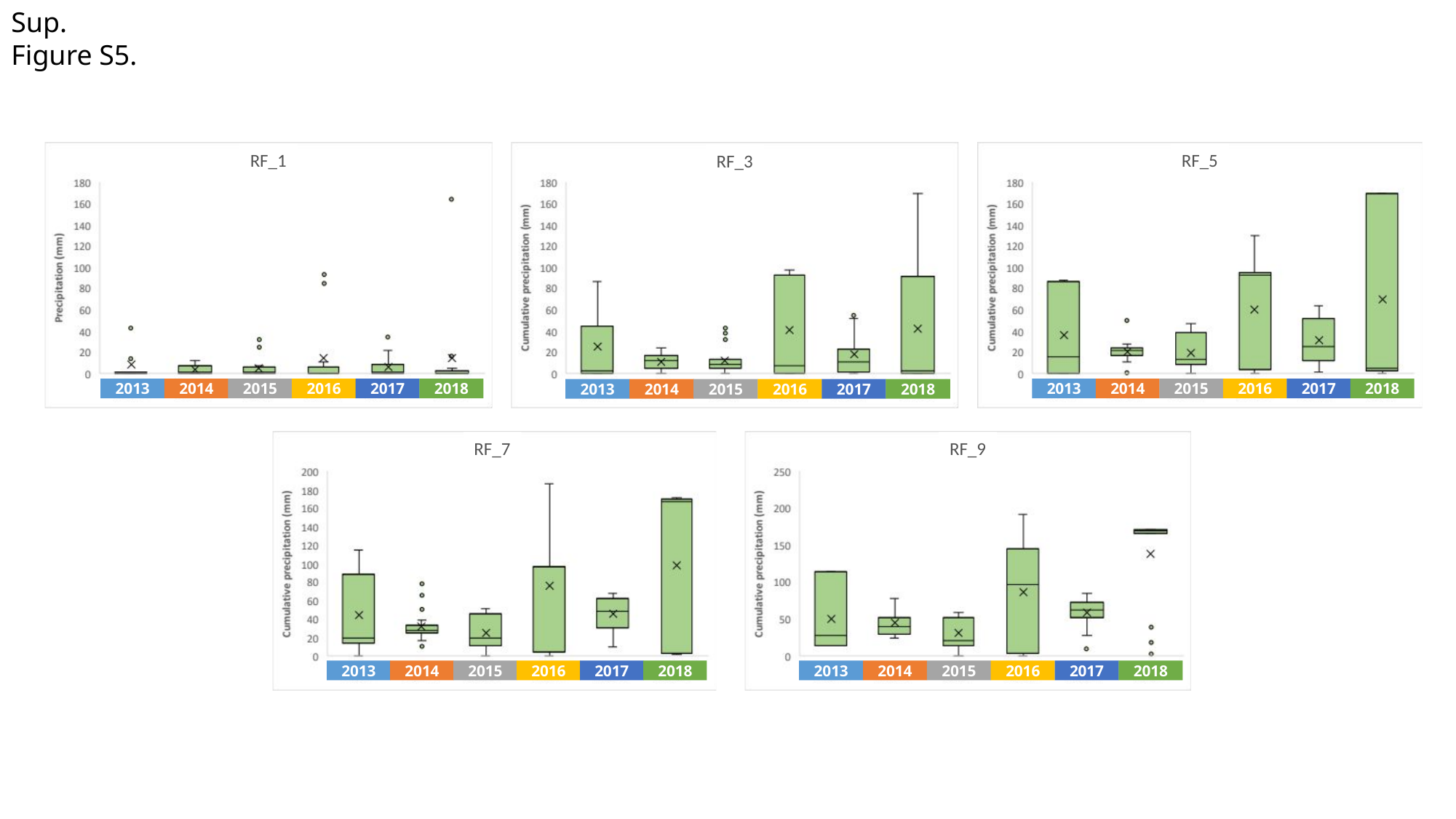

Sup.
Figure S5.
RF_1
RF_5
RF_3
2013
2014
2015
2016
2017
2018
2013
2014
2015
2016
2017
2018
2013
2014
2015
2016
2017
2018
RF_7
RF_9
2013
2014
2015
2016
2017
2018
2013
2014
2015
2016
2017
2018

## Slide 6
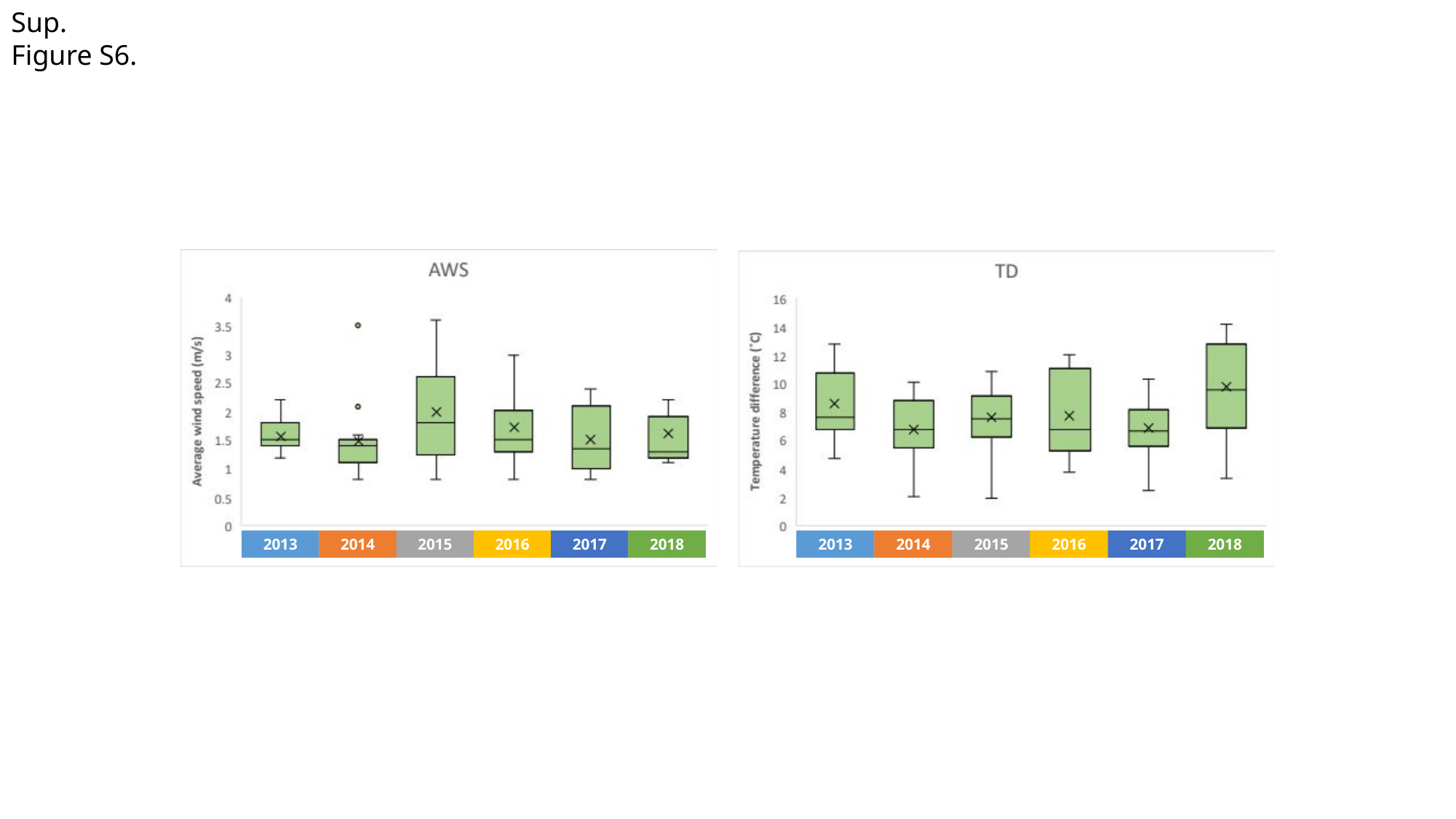

Sup.
Figure S6.
2013
2014
2015
2016
2017
2018
2013
2014
2015
2016
2017
2018

## Slide 7
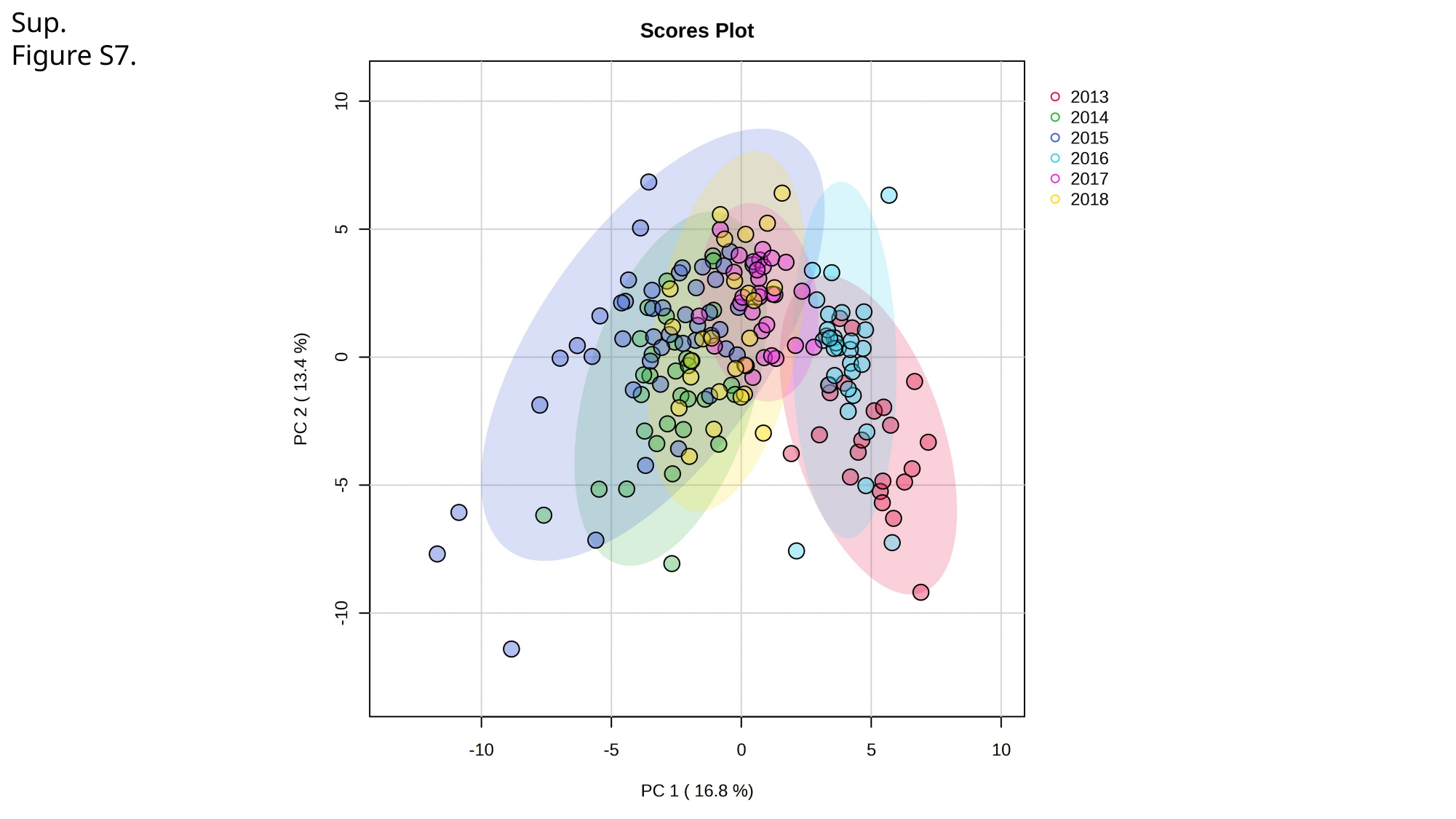

Sup.
Figure S7.
